# Preparation and Anti-Corrosion Performance Investigation of Ni–SiC Composites Produced at Different Ultrasonic Powers

**DOI:** 10.3390/ma18225177

**Published:** 2025-11-14

**Authors:** Lei Qiang, Limei Luo, Mengyu Cao, Xue Guo, Chaoyu Li, Hao Gao

**Affiliations:** 1 School of Mechanical and Electrical Engineering, Sanming University, Sanming 365004, China; 20230338@fjsmu.edu.cn (L.Q.); 20230663205@fjsmu.edu.cn (L.L.); 2 School of Mechanical Science and Engineering, Northeast Petroleum University, Daqing 163318, China; cmy_gx@nepu.edu.cn (M.C.); gx_cmy@nepu.edu.cn (X.G.)

**Keywords:** Ni–SiC composites, electrodeposition, ultrasonic power, microstructure, anti-corrosion performance

## Abstract

To enhance the anti-corrosion performance of storage tanks, Ni–SiC composites were successfully fabricated on the surface of Q345 steel substrate via the ultrasonic electrodeposition technique. The influence of ultrasonic power on the surface morphology, element content, phase structure, and anti-corrosion performance of Ni–SiC composites were explored utilizing a scanning electron microscope (SEM), X-ray diffraction (XRD), energy dispersive spectroscopy (EDS), and an electrochemical workstation, respectively. SEM images showed that the Ni–SiC composites obtained at 120 W had a flat, dense surface morphology, with a uniform distribution of SiC nanoparticles (NPs) and a refined size of nickel grains. Meanwhile, the Si content (7.3 wt.%) of Ni–SiC composites prepared at 120 W was obviously higher than those obtained at 0 W (4.8 wt.%) and 60 W (6.1 wt.%). The thicknesses and adhesion force of Ni–SiC composites manufactured at 120 W were the largest of 103.5 μm and 51.2 N, respectively. XRD patterns presented that the diffraction peaks intensity and width of Ni–SiC composites manufactured at 120 W were lower and broader than that of Ni–SiC composites manufactured at 0 W and 60 W. A corrosion test illustrated that the Ni–SiC composites prepared at 120 W had the lowest corrosion current of 3.5 × 10^−3^ mA/cm^2^, the lowest corrosive weight loss (4.2 mg) and corrosion rate (0.06 mg/h), while the corrosion potential was the highest of −0.41 V, which demonstrated the best anti-corrosion performance. In addition, the co-deposition mechanism of SiC NPs and Ni^2+^ ions was also analyzed.

## 1. Introduction

Storage tanks are popularly applied in agriculture, petroleum, chemical engineering, and civil living. However, the working condition of a high temperature, high pressure, and corrosion medium has limited the service life of storage tanks [1,2,3,4]. Generally, the material of a storage tank is Q345 steel [5]. Enhancing the corrosion resistance of Q345 steel has been essential to extend the usable life of storage tanks.

At present, nickel-based composites (i.e., Ni–SiC, Ni–PTFE, and Ni–Al_2_O_3_) because of strong stability and oxidation resistance, which is considered to be an ideal approach to enhance the corrosion resistance of storage tanks [6,7,8]. In comparison to other nanoparticles, the SiC nanoparticle has the significant advantages of stable physical and chemical property, which has been widely applied in the fields of chemical, electronic, and mechanical engineering [9,10,11]. The SiC nanoparticle has a high melting point and a low heat expansion coefficient, leading to Ni–SiC composites at high temperatures still possessing excellent stability [12]. In addition, the SiC nanoparticle could provide excellent abrasion resistance because of its high hardness value and small friction coefficient, which further enhances the anti-wear performance of coatings [13]. Therefore, these characteristics have resulted in SiC nanoparticles becoming one of the hotspots to improve the synthetic performance of nickel-based composite coatings in harsh environments. Common methods utilized to prepare Ni–SiC composites includes electrodeposition, electroless plating, laser cladding, and thermal spraying [14,15,16,17]. Compared with other fabrication techniques, the electrodeposition’s superior cheap cost, simple operation, and high efficiency have significant advantages [18,19,20]. For instance, Cao et al. [21] investigated the SiC concentration on the microstructure and performance of Ni–SiC composites manufactured via the ultrasonic electrodeposition approach. They proposed that the Ni–SiC composites produced at 7 g/L possessed excellent corrosion resistance and abrasion resistance. Loganathan et al. [22] researched the effect of ultrasonic agitation temperature on the hardness and anti-corrosion performance of electrodeposited Ni–SiC composites. They reported that the Ni–SiC composites prepared at an ultrasonic agitation temperature of 60 °C possessed the highest hardness and outstanding corrosion resistance. Zhang et al. [23] studied the electrodeposited Ni–SiC composites via mechanical stirring and ultrasonic stirring methods. They demonstrated that the introduction of an ultrasonic field contributed to obtaining Ni–SiC composites with a dense, smooth microstructure and an outstanding anti-corrosion performance. He et al. [24] explored the effect of the stirring method on the wear ability and anti-corrosion performance of electrodeposited Ni-W/MWCNTs composites. They proposed the ultrasonic stirring could reduce the agglomeration of MWCNTs and enhance the MWCNTs’s content of composites. These findings demonstrated that the introduction of an ultrasonic field contributed to a decrease in the agglomeration of nanoparticles, resulting in the improved compactness and uniformity of nickel matrix composites. Hence, in this article, the ultrasonic electrodeposition is selected to manufacture Ni–SiC composites.

In the previous literature, some researchers have reported the effect of operation parameters (current density, SiC concentration, magnetic intensity, and jet speed) on the abrasion resistance and anti-corrosion ability of electrodeposited Ni–SiC composites. However, the reports about the influence of ultrasonic power on the anti-corrosion performance of Ni–SiC composites are few. In this paper, the electrodeposited Ni–SiC composites produced different ultrasonic powers. The surface morphology, element content, phase structure, and anti-corrosion performance of Ni–SiC composites were investigated using a scanning electron microscope (SEM), energy dispersive spectroscopy (EDS), X-ray diffraction (XRD), and an electrochemical workstation, respectively. Furthermore, the ultrasonic power impact on the deposition mechanism of electrodeposited Ni–SiC composites was also explored.

## 2. Materials and Methods

### 2.1. Preparation

**Figure 1:**
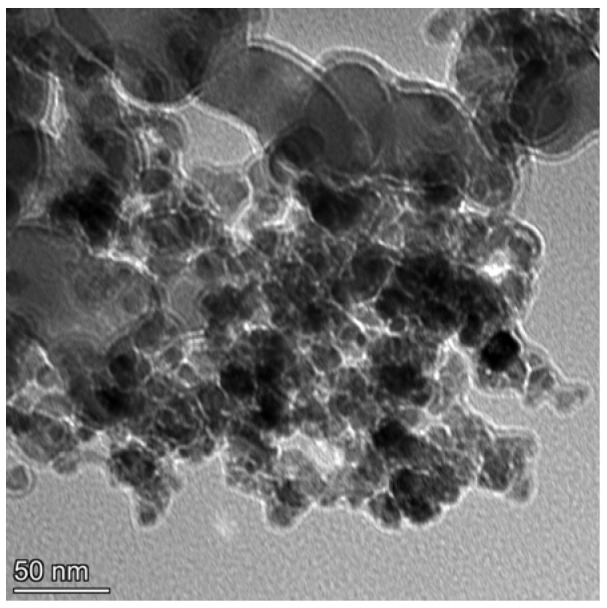
TEM images of a SiC nanoparticle (~20 nm).

Before the ultrasonic electrodeposition process, the nickel sheet at a size of 40 × 30 × 4 mm^3^ acted as an anode, and the Q345 steel substrate at a dimension of 30 × 20 × 3 mm^3^ was employed as a cathode. The TEM image of the SiC nanoparticle is shown in Figure 1. The Q345 steel substrate consisted of Cr (0.25 wt.%), Mn (1.45 wt.%), C (0.15 wt.%), Si (0.33 wt.%), S (0.02 wt.%), Cu (0.21 wt.%), Mo (0.06 wt.%), and balanced Fe. Prior to the electrodeposition process, the substrate was polished, degreased, and activated in sequence. After these treatment steps, the substrate was washed with distilled water and placed in the electrolyte.

**Figure 2:**
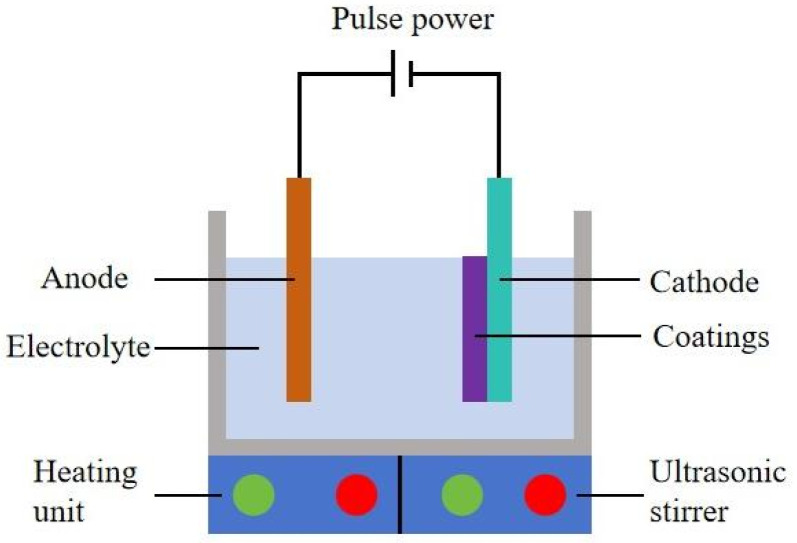
Schematic equipment diagram of Ni–SiC composites manufactured using the electrodeposition approach.

Figure 2 presents the schematic picture of an ultrasonic electrodeposition facility to fabricate Ni–SiC composites. The ultrasonic electrodeposition consisted of a pulse power generator, cathode, anode, ultrasonic stirrer, heating unit, and electrolyte. The ultrasonic stirrer was utilized to produce 60 W and 120 W ultrasonic power for producing Ni–SiC composites. Meanwhile, the Ni–SiC composites manufactured without ultrasonic agitation were defined as 0 W. The heating unit was used to maintain the electrolyte temperature at 48 °C for deposition. The specific chemical components of electrolyte for producing Ni–SiC composites are displayed in Table 1. All of the chemical agents used were of analytical grade.

### 2.2. Characterization

Scanning electron microscopy (SEM, S3400, Hitachi High-tech Corporation, Tokyo, Japan) matched energy dispersive spectroscopy (EDS) were used to observe the surface morphology and chemical composition of Ni–SiC composites. The phase structure of Ni–SiC composites was investigated utilizing an X-ray diffractometer (XRD, D5000, Siemens, Munich, Germany) with an operation condition of Cu kα, scanning speed of 0.02°/s, and scanning range of 30–80°. According to the calculation of Equation (1), the size value of nickel grain size in the Ni–SiC composites could be obtained:

(1) $$D=\frac{0.89\lambda }{\beta \cos\theta }$$

where λ was the wavelength of X-ray, β was the half-width of diffraction peaks, and θ was the angle of Bragg.

To enhance reliability, three specimens were prepared under each condition and averaged for analysis. For the custom cell, the three electrodes electrochemical workstation was employed to measure the anti-corrosion performance of Ni–SiC composites. Meanwhile, the platinum electrode, Ni–SiC composites, and saturated calomel electrode were the counter electrode, working electrode, and reference electrode, respectively. The polarization curves of Ni–SiC composites were obtained at operation parameters of scanning speed (1 mV/s), open-circuit potential (10 mV), and scanning frequency (10^−2^~10^5^ Hz). The adhesion force of Ni–SiC composites was examined using a adhesion tester (TC-A10, Tiandixinghuo Tech-develop Corporation, Beijing, China) under the static pressure time of 30 s, applied force of 100 N, and scratch length of 5 mm. During the corrosion measurement, the contact area of Ni–SiC composites with corrosion liquid (3.5 wt.% NaCl) was set to 1 cm^2^. In addition, to further examine the anti-corrosion performance, the corrosive weight loss (M_c_) of Ni–SiC composites immersed in NaCl solution (3.5 wt.%) for 3 days was calculated using Equation (2):

(2) $$M_{c}=M_{0}-M_{1}$$

where M_0_ and M_1_ were the weight of Ni–SiC composites before and after corrosion measurement, respectively.

Moreover, the corrosion rate (R_c_) of Ni–SiC composites was calculated utilizing Equation (3):

(3) $$R_{c}=M_{c}/t$$

where t was the time of corrosion test.

## 3. Results and Discussion

### 3.1. Surface Morphology Investigation

**Figure 3:**
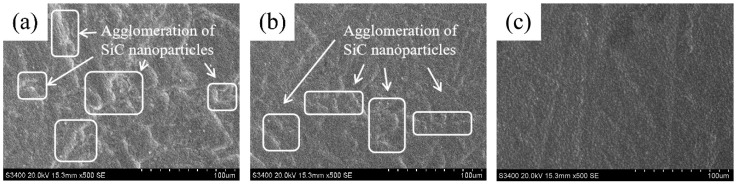
Surface morphology of Ni–SiC composites produced at different ultrasonic powers: (**a**) 0 W, (**b**) 60 W, and (**c**) 120 W.

Figure 3 presents the surface morphology of Ni–SiC composites produced at various ultrasonic powers. The morphology of Ni–SiC composites produced at 0 W consisted of an uneven nickel grain size and obvious agglomeration of SiC nanoparticles (NPs), shown in Figure 3a. After the ultrasonic power increased to 60 W, the morphology defects such as the agglomeration of SiC nanoparticles and variation of nickel grain size that existed on the composite surface reduced significantly. However, the dense and smooth morphology with fine nickel grain size and uniform distribution of SiC NPs appeared on the composite surface prepared at 120 W.

The reason for the above result could be illustrated as follows: (1) The appropriate ultrasonic cavitation could obviously reduce the agglomeration of SiC NPs, leading to a uniform distribution of SiC NPs and generating a strong fine-grain strengthening effect [25]. Meanwhile, the appropriate ultrasonic cavitation was beneficial to decrease the thickness of the diffusion layer, remove the hydrogen bubble absorbed on the substrate surface, and generate the dense and flat surface morphology of the composites [26]. (2) By contrast, the low ultrasonic power caused a weak cavitation effect, leading to the poor fine-grain strengthening effect and not effectively inhibiting the hydrogen evolution reaction [27]. (3) Furthermore, the appropriate ultrasonic cavitation could break large sized grains and nanoparticles, resulting in the grains’ size in the composites being further refined [28]. These reasons combined resulted in the dense, flat morphology that emerged on the surface of the composites obtained at 120 W. The conclusion was similar to the conclusions proposed from Cheng et al. [29] and Li et al. [30].

### 3.2. Element Content Detection

**Figure 4:**
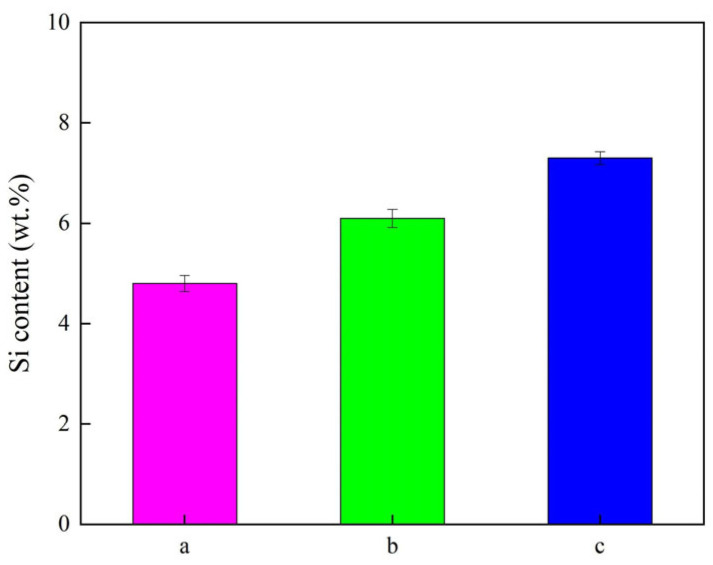
Si content of Ni–SiC composites prepared at various ultrasonic powers: (a) 0 W, (b) 60 W, and (c) 120 W.

The element content (Si) of Ni–SiC composites fabricated at different ultrasonic powers is displayed in Figure 4. The element contents (Si) of Ni–SiC composites prepared at 0 W, 60 W, and 120 W were 4.8 wt.%, 6.1 wt.%, and 7.3 wt.%, respectively. The Si content variation in the Ni-SiC composites could be explained by the introduction of the ultrasonic field contributing to the reduction of the agglomeration of SiC NPs and providing numerous nucleation points, leading to the co-deposition speed of Ni^2+^ ions and SiC NPs. Meanwhile, the appropriate ultrasonic power could generate strong ultrasonic cavitation, which could decrease the surface energy of SiC NPs and enhance the co-deposition speed of Ni^2+^ ions and SiC NPs. This result has been demonstrated by the similar conclusion reported by Li et al. [31] that found the relationship of ultrasonic power impacted the Si content of Ni–SiC composites manufactured using the electrodeposition approach. Therefore, the element content (Si) of Ni–SiC composites deposited at 120 W was higher than those obtained at 0 W and 60 W.

### 3.3. Composites Thickness Analysis

**Figure 5:**
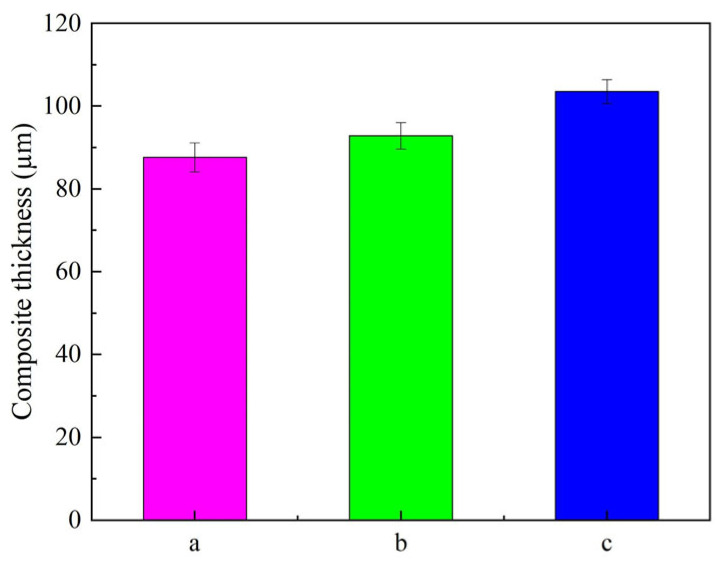
Thickness value of Ni–SiC composites manufactured at different ultrasonic powers: (a) 0 W, (b) 60 W, and (c) 120 W.

The thickness value of Ni–SiC composites manufactured at various ultrasonic powers is revealed in Figure 5. The thickness value of Ni–SiC composites prepared at 0 W was only 87.6 μm. After the ultrasonic power rose to 60 W, the thickness value of Ni–SiC composites increased to 92.8 μm. However, the thickness value of composites produced at 120 W was the largest, which reached up to 103.5 μm.

**Figure 6:**
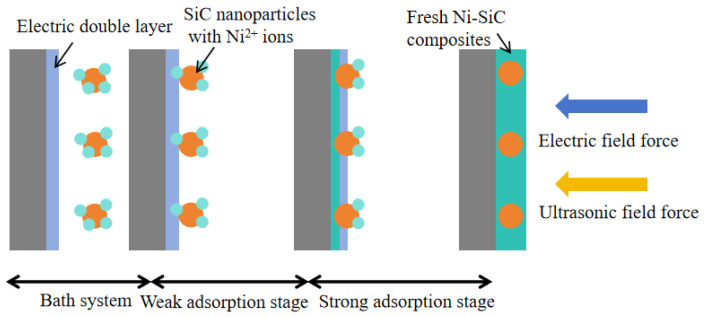
Co-deposition mechanism of SiC NPs and Ni^2+^ ions.

The variation in thickness could be illustrated by the co-deposition mechanism of SiC NPs and Ni^2+^ ions, shown in Figure 6. The co-deposition mechanism could be clearly illustrated using the absorption model from Gugliemi. During the electrodeposition process, the nickel grains grew rapidly due to the absence of an ultrasonic field, resulting in the formation of uneven nickel grain size and obvious agglomeration of SiC NPs [32]. By comparison, the introduction of ultrasonic power caused the reduction in SiC NPs agglomeration in the electrolyte. Importantly, under the function of the electric field force and appropriate ultrasonic field, the SiC nanoparticle with Ni^2+^ ions deposited on the surface of the substrate and formed Ni–SiC composites. The plentiful SiC NPs could provide numerous nucleation points, which significantly inhibited the quick growth of nickel grains. Furthermore, high ultrasonic power could obviously decrease the thickness of the electric double layer and accelerate the co-deposition speed of Ni^2+^ ions and SiC NPs. This effect had more obvious influence on the composites fabricated at 120 W, where the appropriate ultrasonic power contributed to enhance the co-deposition speed and obtain better dispersion of SiC nanoparticles (Seen in Figure 3b,c). A similar conclusion has been reported by Chen et al. [33].

### 3.4. XRD Pattern Examination

**Figure 7:**
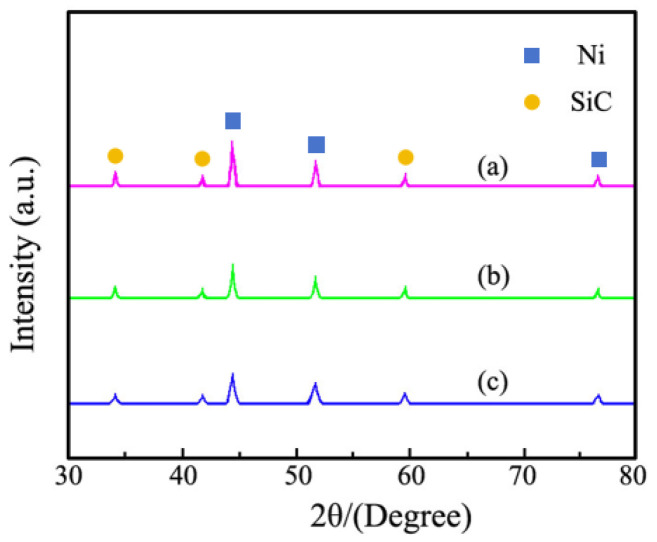
XRD spectrum of Ni–SiC composites prepared at various ultrasonic powers: (a) 0 W, (b) 60 W and (c) 120 W.

The XRD pattern obtained from Ni–SiC composites manufactured at different ultrasonic powers are revealed in Figure 7. The XRD pattern results demonstrated that SiC NPs existed in all Ni–SiC composites. A high intensity peak of composites at 43.8° and two low intensity peaks of composites at 52.7° and 75.3° were assigned to the nickel crystal planes of (111), (200), and (220) with face-centered cubic structure, respectively [34]. Meanwhile, characteristic diffraction peaks of SiC NPs were found at 34.3°, 41.5°, and 59.8°, corresponding to the crystal planes of (111), (200), and (220), respectively [35]. Moreover, the diffraction peak intensities and width of composites became lower and wider with the increasing ultrasonic power. Based on the computation of Equation (1) [36], the nickel grain size of Ni–SiC composites prepared at 0 W, 60 W, and 120 W were 417.3 nm, 263.8 nm, and 178.5 nm, respectively. The result illustrated that the high ultrasonic power was beneficial to reduce agglomeration of SiC NPs and enhance fine-grain strengthening effect, which led to grain size significantly decreasing. In addition, the high ultrasonic power generated a strong ultrasonic cavitation effect, which could crush the large sized grains and decrease the grain size. These conclusions confirmed the findings of Cao et al. [37], who proposed that the relationship between ultrasonic power and the property of nickel matrix composite coatings could be fabricated using the electrodeposition technique.

### 3.5. Adhesion Force Detection

**Figure 8:**
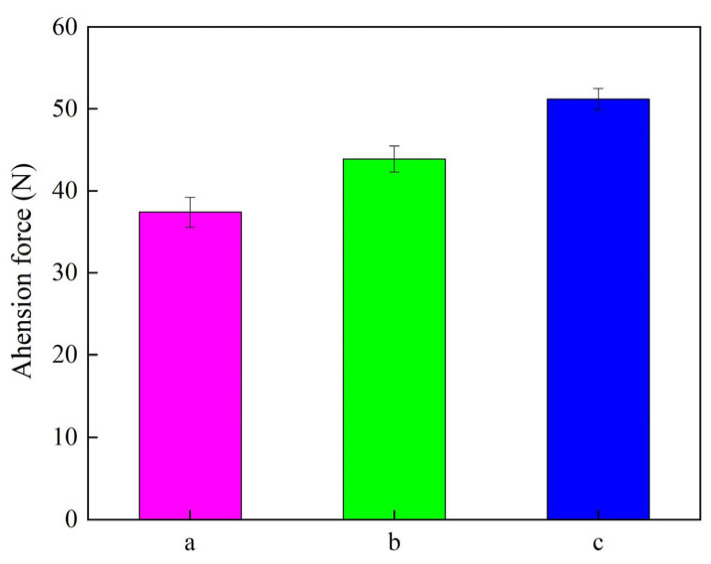
Adhesion force diagram of Ni–SiC composites fabricated at various ultrasonic powers: (a) 0 W, (b) 60 W, and (c) 120 W.

The adhesion force diagram of Ni–SiC composites obtained at different ultrasonic powers is displayed in Figure 8. The adhesion forces of Ni–SiC composites produced at 0 W, 60 W, and 120 W were 37.4 N, 43.9 N, and 51.2 N, respectively. The reason for this result could be illustrated as follows: (1) The introduction of the ultrasonic field acted as mechanical agitation and caused the occurrence of grain refinement, leading to the bonding force of Ni–SiC composites deposited on the substrate surface increased. (2) Moreover, the appropriate ultrasonic power contributed to the decrease in the internal stress of composites, which resulted in the adhesion force of Ni–SiC composites being further improved. These findings were similar to the conclusion of Ji et al. [38].

### 3.6. Anti-Corrosion Performance Measurement

**Figure 9:**
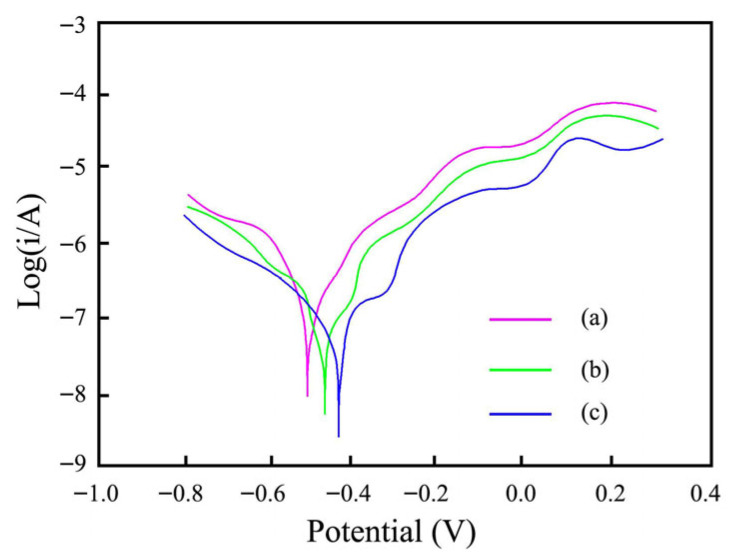
Polarization curves picture of Ni–SiC composites fabricated at various ultrasonic powers: (a) 0 W, (b) 60 W, and (c) 120 W.

The polarization curves from Ni–SiC composites produced at different ultrasonic powers are displayed in Figure 9. The measured conditions were conducted at 3.5 wt.%, in NaCl solution, and at room temperature. The measured corrosion potential (Ecorr) from Ni–SiC composites deposited at 0 W, 60 W, and 120 W assigned to −0.52 V, −0.46 V, and −0.41 V, respectively. However, the measured corrosion current density (Icorr) from Ni–SiC composites manufactured at 0 W, 60 W, and 120 W were 7.8 × 10^−3^ mA/cm^2^, 5.1 × 10^−3^ mA/cm^2,^ and 3.5 × 10^−3^ mA/cm^2^, respectively. Li et al. [39] and Zhang et al. [40] found that the high Ecorr and low Icorr of nickel matrix composites possessed excellent anti-corrosion performance. In comparison to other composites, the Ni–SiC composites obtained at 120 W had the lowest Icorr and the highest Ecorr, indicating the best anti-corrosion performance.

**Figure 10:**
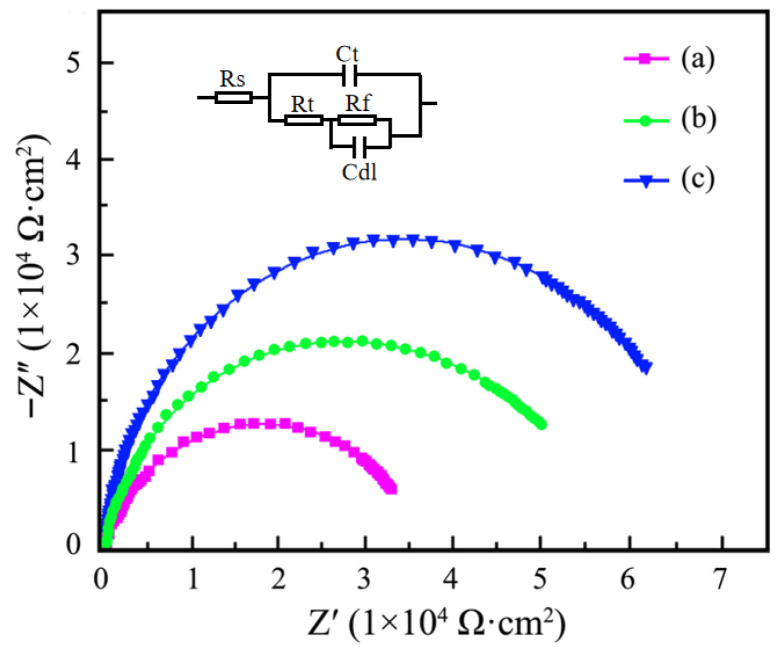
Nyquist plots and equivalent circuit picture of Ni–SiC composites prepared at various ultrasonic powers: (a) 0 W, (b) 60 W and (c) 120 W.

The relationship of ultrasonic power impact on the Nyquist plots of Ni–SiC composites is depicted in Figure 10. In the equivalent circuit picture, Cdl, Rs, Rf, Rt, and Ct were the capacitance of Ni–SiC composites, the corrosion liquid resistance, the charge transfer resistance, the relevant chemical reaction of charge transfer, and the double-layer diffusion, respectively. The impedance value of Ni–SiC composites produced at 0 W was the lowest, demonstrating a bad anti-corrosion resistance. After the ultrasonic power grew to 60 W, the impedance value of Ni–SiC composites increased, demonstrating the anti-corrosion performance was enhanced. However, among three Ni–SiC composites, the one produced at 120 W had the largest impedance value, illustrating an excellent anti-corrosion performance. Li et al. [41] and Nasri et al. [42] proposed that the impedance value could effectively reflect the anti-corrosion performance of nickel matrix coatings manufactured using electrodeposition. The nickel matrix composites had a large impedance value, which demonstrated an outstanding anti-corrosion performance. Hence, the anti-corrosion performance of Ni–SiC composites deposited at 120 W was superior to those produced at 0 W and 60 W.

**Figure 11:**
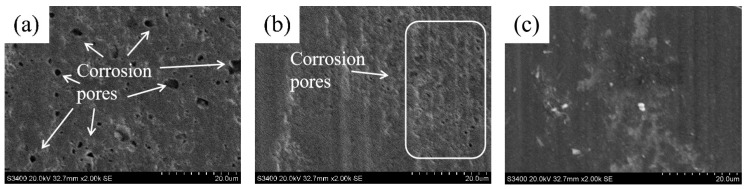
Corrosive surface morphology of Ni–SiC composites produced at various ultrasonic powers: (**a**) 0 W, (**b**) 60 W, and (**c**) 120 W.

The corrosive surface morphology of Ni–SiC composites fabricated at different ultrasonic powers are illustrated in Figure 11. The average corrosive weight loss and corrosion rate of Ni–SiC composites are listed in Table 2. The corrosion measurement results show that numerous and large corrosion pits emerged on the surface of composites prepared at 0 W. After the ultrasonic power increased to 60 W, the size and quantity of corrosion pits that existed on the surface of Ni–SiC composites reduced greatly. However, the obvious corrosion pits were not found on the surface of composites produced at 120 W. According to Equation (2), the average corrosive weight loss of Ni–SiC composites produced at 0 W, 60 W, and 120 W were 7.9 mg, 6.3 mg, and 4.2 mg, respectively. Based on Equation (3), the corrosion rate of Ni–SiC composites manufactured at 0 W, 60 W, and 120 W were 0.11 mg/h, 0.09 mg/h, and 0.06 mg/h, respectively.

The results caused by the above phenomenon could be attributed to the varying surface morphology, different contents, and distribution of SiC NPs. In comparison to the other two Ni–SiC composites, the one obtained at 120 W possessed dense surface morphology, which could significantly hinder the destruction of the NaCl solution [43]. Meanwhile, the anti-corrosion performance of SiC NPs was superior to that of nickel, which caused the anti-corrosion performance of composites with high SiC NPs content to be excellent [44]. Furthermore, the SiC NPs of Ni–SiC composites fabricated at 120 W were plentiful and even-distributed, which could immensely prolong the corrosion path of NaCl solution [45]. Therefore, the anti-corrosion performance of Ni–SiC composites produced at 120 W indicated the outstanding anti-corrosion performance.

## 4. Conclusions

(1) Surface morphology presented the Ni–SiC composites produced at 0 W and 60 W had uneven nickel grain size and obvious agglomeration of SiC NPs. By comparison, the dense, smooth morphology with fine nickel grain size and uniform distribution SiC NPs appeared on the surface of Ni–SiC composites produced at 120 W. Furthermore, the Si contents of Ni–SiC composites prepared at 0 W, 60 W, and 120 W were 4.8 wt.%, 6.1 wt.%, and 7.3 wt.%, respectively.

(2) The thicknesses and adhesion force of Ni–SiC composites manufactured at 120 W were the largest of 103.5 μm and 51.2 N, respectively. XRD patterns revealed that SiC NPs were deposited in three Ni–SiC composites. Moreover, the diffraction peak intensities and width of Ni–SiC composites became lower and wider with the increasing ultrasonic power.

(3) In comparison to the other two Ni–SiC composites, the one produced at 120 W had the highest Ecorr of −0.41 V and the lowest Icorr of 3.5 × 10^−3^ mA/cm^2^, indicating the anti-corrosion performance was outstanding. In addition, the average corrosive weight loss and corrosion rate of Ni–SiC composites manufactured at 120 W was much lower compared to those obtained at 0 W and 60 W, illustrating the best anti-corrosion performance.

## Figures and Tables

**Table 1:** The plating condition and composition of electrolytes used to prepare Ni–SiC composites.

Operation Parameters	Specific
Current density (A/dm^2^)	5
Plating temperature (°C)	48
Electrodeposition time (min)	50
NiSO_4_ (g/L)	170
NiCl_2_ (g/L)	25
pH value	4.7
SiC concentration	5
CTAB (mg/L)	50
H_3_BO_3_ (g/L)	40

**Table 2:** Average corrosive weight loss and corrosion rate of Ni–SiC composites fabricated at different ultrasonic powers.

Ni–SiC Composites	Average Corrosive Weight Loss	Corrosion Rate (mg·h^−1^)
Prepared at 0 W	7.9 mg	0.11
Prepared at 60 W	6.3 mg	0.09
Prepared at 120 W	4.2 mg	0.06

## Data Availability

The original contributions presented in this study are included in the article. Further inquiries can be directed to the corresponding authors.
